# Reasons for Tooth Extractions and Related Risk Factors in Adult Patients: A Cohort Study

**DOI:** 10.3390/ijerph17072575

**Published:** 2020-04-09

**Authors:** Pier Carmine Passarelli, Stefano Pagnoni, Giovan Battista Piccirillo, Viviana Desantis, Michele Benegiamo, Antonio Liguori, Raffaele Papa, Piero Papi, Giorgio Pompa, Antonio D’Addona

**Affiliations:** 1 Department of Head and Neck, Division of Oral Surgery and Implantology, Institute of Clinical Dentistry, Università Cattolica del Sacro Cuore, Fondazione Policlinico Universitario Gemelli, 00168 Rome, Italy; stefano_pagnoni@yahoo.com (S.P.); giovanbpiccirillo@gmail.com (G.B.P.); desantisviviana@gmail.com (V.D.); michelebenegiamo@hotmail.it (M.B.); raffaele.papa@gmail.com (R.P.); antonio.daddona@policlinicogemelli.it (A.D.); 2 Internal Medicine Department, Fondazione Policlinico A. Gemelli, Catholic University of Sacred Heart, 00168 Rome, Italy; lig.antonio91@gmail.com; 3 Department of Oral and Maxillo-Facial Sciences, “Sapienza” University of Rome, 00185 Rome, Italy; piero.papi@uniroma1.it (P.P.); giorgio.pompa@uniroma1.it (G.P.)

**Keywords:** dentistry, extractions, oral health, caries

## Abstract

*Background*: The aim of this study was to evaluate oral status, the reasons for tooth extractions and related risk factors in adult patients attending a hospital dental practice. *Methods*: 120 consecutive patients ranging from 23 to 91 years in age (mean age of 63.3 ± 15.8) having a total of 554 teeth extracted were included. Surveys about general health status were conducted and potential risk factors such as smoking, diabetes and age were investigated. *Results*: a total of 1795 teeth were missing after extraction procedures and the mean number of remaining teeth after the extraction process was 16.8 ± 9.1 per patient. Caries (52.2%) was the most common reason for extraction along with periodontal disease (35.7%). Males were more prone to extractions, with 394 of the teeth extracted out of the total of 554 (71.1%). Male sex (β = 2.89; 95% CI 1.26, 4.53; p = 0.001) and smoking habit (β = 2.95; 95% CI 1.12, 4.79; p = 0.002) were related to a higher number of teeth extracted. Age (β = −0.24; 95% CI −0.31, −0.16; p < 0.001) and diabetes (β = −4.47; 95% CI −7.61, −1.33; p = 0.006) were related to a higher number of missing teeth at evaluation time. Moreover, periodontal disease was more common as a reason of extraction among diabetic patients than among non-diabetic ones (p = 0.04). *Conclusions*: caries and periodontal disease were the most common causes of extraction in a relatively old study population: further screening strategies might be required for the early interception of caries and periodontal disease.

## 1. Introduction

Tooth loss is one of the main indicators of oral health in a population and is one of the favorite variables considered in much research [1]. The study of missing teeth in a population can be useful to determine whether the level of oral hygiene is good or if oral care is adequate and accessible [2,3]. An evaluation of proper hygiene education and environment-related issues can be made when comparing the dental records of different population groups. Many factors can be used to compare similar populations, and a vast number of records are available today to assess oral health levels within a community [4,5,6,7,8,9,10]. Some of the most useful variables are common wealth, nationality, the belonging to an ethnic group, age and employment.

General health status, systemic disease [11], such as metabolic syndromes, syndromic diseases [11,12,13] causing dental abnormalities and immune dysfunction issues can lead to severe complications and poor oral conditions [14]. Within a population, there are significant differences between groups of different ages and employment. It is very important, when analyzing and comparing data, to differentiate the results, taking these variables into account and to consider the hypothesis of splitting those records into more homogenous groups to highlight the causes of tooth loss and possible solutions. By identifying tooth loss, its main causes and its related risk factors, it may be possible to limit future extractions and highlight the crucial role of prevention in reducing caries and periodontal pathology. It is important to understand the consequences of tooth loss on dentition, oral functions and possibilities for future treatments: after the extraction of a tooth, the alveolar ridge undergoes progressive bone resorption, and this can lead to functional and aesthetic deficits to the patient [15,16]. Since 1970, many preventive measures were taken to reduce teeth loss incidence to manageable proportions. Such preventive measures have included public water fluoridation and the spreading of fluoride-containing toothpastes. Tooth loss in younger individuals is often due to poor hygiene education and self care [17]. Tooth loss events lead to substantial direct costs for tooth replacement and should be an appropriate outcome for the effectiveness of long-term oral disease prevention measures [18].

The aim of the study was to evaluate general oral health conditions in an adult population, considering many factors such as the number of extracted teeth, number of missing teeth after surgical procedures and reasons for extractions, seeking potential correlations between tooth loss and several variables including age, gender, education and other risk factors such as smoking habit and diabetes.

## 2. Materials and Methods

This retrospective study investigates data (clinical charter and radiological documentation) of all 210 adult patients who were referred to the Oral Surgery Department, Policlinico Universitario Agostino Gemelli (Rome, Italy) between September and December 2017. Patients visited our Department because they were being treated at the Gemelli Hospital Dental Clinic, that refers us patients needing extraction procedures. The population attending our department is mixed, although most of our patients suffer low socio-economic status (annual household income < 10,000 €), since dental care is free of charge for them in our hospital. Patients were excluded if they were suffering uncompensated systemic conditions, if they presented an American Society of Anesthesiologists’ physical status classification of IV, or if coming from accidents and trauma.

Patients diagnosed with mild-to-severe mental disorders are not included in our study group. Such patients are treated in a different Dental Unit. Mental disorders, anxiety and depression are commonly related to bad oral health conditions [19], hence the need for a separated Dental Unit for special-needs patients.

We also excluded from our analysis patients who did not undergo tooth extraction after our visit; the causes were the patient’s refusal, a decision for a restorative treatment in a compromised tooth, and missing appointments. One hundred and twenty patients were finally included in the study. All of the patient’s data (including age, sex, medical history, dental check-up frequency, scholar education level, oral hygiene education level [20], the use of fluoride toothpaste, cigarette consumption, comorbidities, pharmacological therapy, cause for extraction, number of teeth before and after the extraction procedure and post-extraction Kennedy class) were recorded in a standard form filled out by a single practitioner (AD) who interviewed and visited each patient before the extraction procedures; the causes for extraction were carefully evaluated by this single trained operator (AD), who visits patients routinely in our unit, before any extraction appointment is given to the patient. If there was more than one cause for extraction, we reported the most major one.

A panoramic radiograph was obtained and analyzed to define a proper treatment plan, and it was executed with a digital method that showed a greater diagnostic precision [21].

A patient-specific evaluation was carried out each time to make an evidence-based indication suitable for each patient. Patients’ clinical conditions varied as in the general population, and we considered all of the variables for each patient who underwent extraction procedures. A patient was considered to have a smoking habit if either they were an active smoker at the time of evaluation or they had been a smoker during the past 10 years.

Teeth with a pulpal pathology were managed by endodontic therapy where possible, although teeth with recurrent periapical infection or insufficient coronal tooth structure for restoration were extracted.

A periodontal exam was performed; sometimes, a severe periodontal disease may lead to tooth extraction [22].

Because of the retrospective nature of the present study, it was granted an exemption in writing by the institutional review board of the Catholic University of Sacred Heart of Rome. An informed consent form was obtained from all patients. We read the Declaration of Helsinki and followed the guidelines in this investigation.

### Statistical Analysis

Data regarding 554 teeth extracted from a sample of 120 patients were recorded and analyzed by statistical analysis software (STATA14) (StataCorp LLC, College Station, TX, USA). All statistical tests were two-tailed using a 0.05 level of significance. Continuous variables were summarized as mean ± standard deviation, and categorical variables as frequency and percentage.

The chi-squared test was used to compare categorical variables. Student’s *t*-test was used to evaluate the distribution of continuous variables stratified for categorical variables (tests for the assessment of the normality of the distribution of continuous variables were previously used). Pearson’s R coefficient was used to compare continuous variables.

Simple and multiple linear regression models were performed, considering the number of teeth extracted or the number of teeth at the time of evaluation as a dependent variable, and sex, age, diabetes and smoking habit as independent variables. The results of the linear regression models are expressed as coefficient β, confidence interval, p-value. R-squared is shown as an expression of how much variation of the dependent variable is explained by the independent variables forced in regression models.

## 3. Results

### 3.1. Descriptive Statistics

The mean age of enrolled patients was 63.3 ± 15.8, ranging from 23 to 91 years. Most of the patients included were male (55.8%), and people with a high school education were the most common (34.2%), whereas only a few patients had graduated from a university (10.8%). A great number of them did not attend a dentist for a regular checkup yearly, with more than 70% of the patients waiting more than a year between two medical examinations (Table 1).

All of the patients enrolled used fluoride toothpaste regularly, according to the data collected by the dentist before any procedure.

Of the patients, 95.8% came to our examination with one or more teeth already missing, whereas the mean number of in arch teeth at the time of examination was 21.5 ± 7.5.

The mean number of extracted teeth for each patient was 4.6 ± 4.7, and the mean number of remaining teeth after the extraction process was 16.8 ± 9.1.

Kennedy’s classification was used to determine the pattern of partially edentulous arches [23]; Kennedy Class 3 was the most common for both the upper and lower jaws, while the least common was Class 4, with only one patient for each group presenting only posterior sextants after the procedure.

A total of 33 patients (27.5%) had smoking habits and 27 (22.5%) were affected by compensated diabetes. Seven patients (5.8%) had both smoking habits and diabetes.

### 3.2. Sex, Diabetes and Smoking Habit as Key Factors Determining Dental Status

In Table 2 are shown results obtained from stratifying the entire population of included patients by sex, smoking habits and diabetes, which are known to be risk factors for tooth decay and extraction [24,25]. In our population, patients affected by diabetes had a higher mean age (p < 0.05), a lower educational level (p < 0.05) and a lower number of teeth both before (p < 0.05) and after (p < 0.05) the evaluation, while no significant difference was found in the number of teeth extracted at the time of evaluation.

The number of teeth extracted was significantly higher in patients with smoking habits (p < 0.05), but there was no difference considering the number of teeth before and after the evaluation.

The number of teeth extracted was significantly higher in male patients (p < 0.001), with no significant difference in the number of teeth before evaluation between males and females.

**Figure 1:**
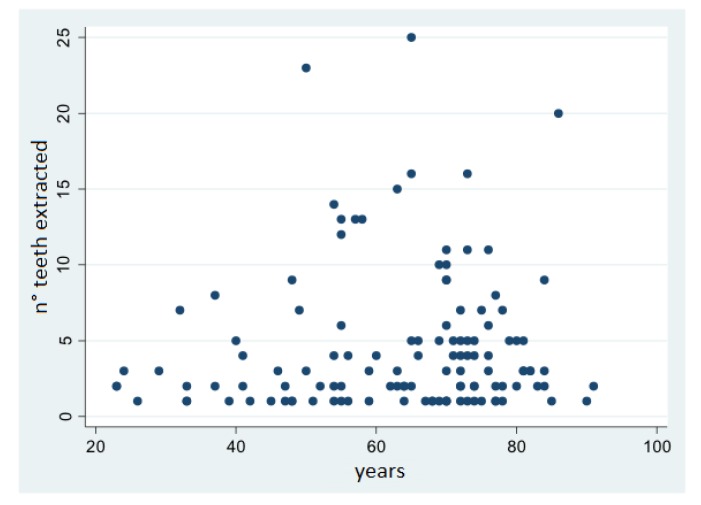
Correlation between age and number of teeth extracted.

**Figure 2:**
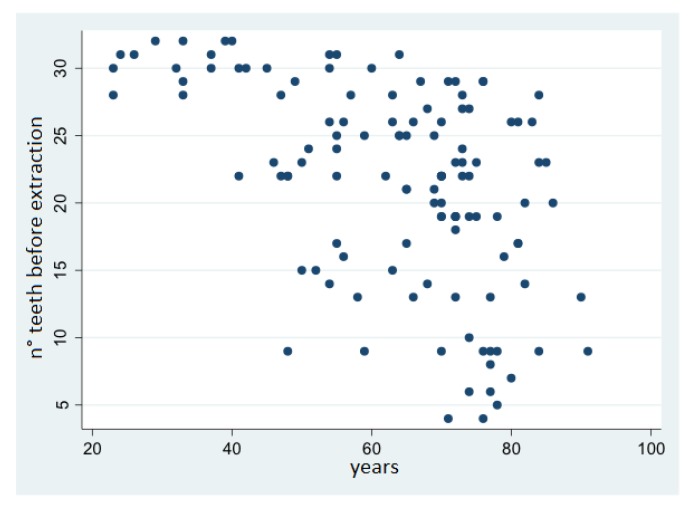
Correlation between age and number of teeth before extraction.

The association between age and patients’ dental status was explored and is shown in the scatterplots in Figure 1 and Figure 2. There was a rather strong association between age and the number of teeth before extraction (Pearson R = −0.51) and after extraction (Pearson R = −0.45), while there was a weak association between age and the number of teeth extracted (Pearson R = 0.07).

### 3.3. Factors Associated with the Number of Teeth Extracted and the Number of Teeth before Extraction

Linear regression models were performed to evaluate factors associated with the number of teeth extracted and factors associated with the number of teeth at the time of evaluation (Table 3). In univariate linear regression models, there was a significative association between male sex and the number of teeth extracted (β = 2.89; 95% CI 1.26, 4.53; p = 0.001) and between smoking habit and the number of teeth extracted (β =2.95; 95% CI 1.12, 4.79; p = 0.002), while diabetes and age were not significantly associated with the number of teeth extracted. In the multiple linear regression model, smoking habit (coefficient 2.47; 95% CI 0.67, 4.27; p = 0.008) and male sex (coefficient 2.51; 95% CI 0.89, 4.13; p = 4.13) remained independently associated with the number of teeth extracted (multiple linear model R^2^ = 0.15).

In univariate linear regression models, there was a significative association between age and the number of teeth before extraction (β = −0.24; 95% CI −0.31, −0.16; p < 0.001) and between diabetes and the number of teeth before extraction (β = −4.47; 95% CI −7.61, −1.33; p = 0.006). Male sex and smoking habits were not significantly associated with the number of teeth before extraction (Table 3).

In the multiple linear regression model, the coefficient result for age was −0.22 (95% CI −0.30, −0.15; p < 0.001), and for diabetes was −2.19 (95% CI −5.07, 0.70; p = 0.14), with R^2^ = 0.27.

### 3.4. Causes for Tooth Extraction

Five hundred and fifty-four (554) teeth have been extracted. Molars were the most commonly extracted teeth (n = 210, 37.9%) and canines were the least common ones (n = 71, 12.8%). Lower teeth were slightly more prone to extraction (50.4%). A total of 1795 teeth were missing after the surgical procedures, with most of them being first and third molars (respectively 18.2% and 19.6%), while the upper central incisors (3%) and lower canines (2.7%) were the fewest.

Dental caries and periodontal disease were the main causes for extraction, with 289 teeth (52.2%) lost due to caries and 198 teeth (35.7%) lost due to periodontal disease; only 38 teeth (6.9%) were lost due to endodontic issues, while 16 teeth (2.9%) were removed for prosthetic indications and 13 teeth (2.3%) were lost due to the failure of previous treatments (Table 4).

In our population, tooth extraction for periodontal disease was more common in diabetic patients than in non-diabetic ones (p = 0.04), while the distributions of the causes of tooth extraction were not statistically different between smokers and non-smokers (Table 4). A total of 44 teeth were extracted from smokers and diabetic patients; 50% of teeth were extracted due to periodontal disease, while 45.4% were extracted due to caries, with no significant differences between non-diabetic and non-smoking patients (Table 4).

Of all of the molars, 54.6% were extracted for caries, and only one third of the molars had to be extracted due to periodontal disease. Incisors were most commonly extracted due to periodontal issues (48% of all extracted incisors).

## 4. Discussion

Interesting results are obtained when the patient’s age is considered for each cause of tooth extraction. Tooth loss due to periodontal disease is common in elderly patients with a mean age of 67.6 ± 11.5. Caries are common in a slightly younger population with a mean age of patients undergoing tooth extraction of 63.0 ± 14.9, while endodontic issues leading to extraction are common in an even younger population with a mean age of 61.8 ± 12.2.

Many articles show a similar correlation with overlapping results: in Italy, Norway, Greece, England and Wales, Kuwait and Japan, periodontal disease is most common in elderly populations, while caries is the main cause for extraction and affects many individuals, being related to not only age but also to education and socio-economic status [4,5,7,26,27,28,29,30].

Other articles show that the main cause for tooth loss is periodontal disease [31,32]; this could be due to differences in treatment planning, demographic distribution, age, diet and education.

Similar studies collect most of the data from private practitioners, whereas our study was conducted in a hospital environment, thus showing results for a less wealthy population with poor education and self care [15].

Despite having different education levels, all of the patients included in the study were using standard 1450 ppm fluoride concentrations; however, cavities remain the major problem in a proportion of the population. Regular dental visits are still not a routine pattern of behavior for all. Diagnosing caries at an early stage can prevent extensive dental treatment and teeth extraction. Furthermore, strategies for efficient ways of screening for caries and periodontal disease, especially for middle-aged people, might be required.

Moreover, the mean age of the population in our study was relatively high (63.3 ± 15.8 years) compared to those in other studies, ranging from 23 to 91 years of age, showing no data of tooth extraction for orthodontic issues [5,8,33,34,35].

A mean of 4.6 ± 4.7 teeth per patient were lost during extraction procedures. This number is high compared to in other studies in Japan (1.53 teeth per patient), Canada (2.3 teeth per patient), Kuwait (1.73 teeth per patient) and Norway (1.3 teeth per patient), being much higher than the mean number of extracted teeth in Italy recorded by Angelillo et al. [5] (1.77 for patients >65 years old) [5,7,9,28,30]. The results may be explained by the fact that a large number of the people treated in the hospital come from a low socio-economic status. Tooth loss is often due to poor self care in high-risk individuals that have no access to disease prevention and early diagnosis. In fact, 85 of the 120 patients enrolled had not attended a dentist for over a year at the time of extraction, 115 (95.8%) of them came to our examination with at least one tooth already missing, and the mean number of in-arch teeth before the extraction process was 21.5 ± 7.5, showing a critical situation for most of the patients. The mean number of in-arch teeth after the extraction process was 16.8 ± 9.1, with the patients most commonly presenting a Kennedy Class 3 dentate situation with unilateral bounded posterior saddles.

Incisors were most commonly extracted due to periodontal issues (48% of all extracted incisors), probably because they are less prone to developing cavities and are most commonly lost by old patients affected by periodontal disease [6].

Molars were most commonly extracted due to caries (54.6% of all of the molars extracted), and only one third of them had to be extracted due to periodontal disease. Molars are more prone to developing cavities because of their anatomy, marked by pits and grooves. Poor oral hygiene leads to the early loss of first and second molars.

Our study was conducted on a relatively old population, but differences in the mean age of patients undergoing extraction of different teeth are clear; our study reported that the mean age for incisor loss was 67.9 years, while patients losing first and second molars were 60.7 years old on average.

Of the 120 enrolled patients, a total of 67 (55.8%) were male, and of the 554 teeth extracted, 396 (71.1%) were extracted from male patients, thus showing how males were more prone to multiple extractions than females. Some authors think this happens because males are less interested in reconstructive therapies than females [6].

Our study did not show differences in the causes for extractions between genders. Proportionally, the same percentage of teeth were extracted due to periodontal disease and caries for both sexes (periodontal disease: 36% and 33% in males and females respectively; caries: 52.2% and 50% in males and females respectively). Thus, our study disagrees with others showing that gender could be a risk factor for periodontal disease, reporting a higher percentage of teeth lost due to periodontal disease in males than in females [4,6,36].

Smoking habit or diabetes alone does not influence the proportion of teeth lost due to periodontal disease or caries (Table 4), but when considering the total of 44 teeth extracted from patients with both diabetes and a smoking habit, periodontal disease was most common, responsible for 50% of the teeth extracted, while only 47.7% were extracted due to caries. These results show how diabetes and cigarette smoke, combined, could contribute to severe periodontal disease.

Our results highlight oral hygiene problems affecting low-income populations attending in-hospital dental care. Some authors [5] found much better oral care during private practice experiments when examining the causes of extraction.

The results show how needy populations are also more exposed to oral disease and less sensitive to general self care.

## 5. Conclusions

Caries and periodontal disease were the most common causes of extraction in a relatively old study population.

Smoking habit and sex were found to be good predictors for the number of extracted teeth, while diabetes and age were not associated with a higher number of extractions. Further studies are needed to provide an overview of the oral healthcare status of in-hospital populations. Most studies are conducted by private practitioners, often showing optimistic results regarding the matters of the numbers of teeth lost and oral disease. Further efficient screening strategies for caries and periodontal disease, especially for middle-aged people, might be required for prevention.

## Figures and Tables

**Table 1:** The demographic and clinical features of the study population.

Total Population n = 120 Age: 63.3 ± 15.8 - Sex [males]: 67 (55.8)			
Education level		Time of last dental visit	
Elementary school	31 (25.8)	6 months	7 (5.8)
Middle school	35 (29.2)	1 year	28 (23.5)
High school	41 (34.2)	More than 1 year	85 (70.8)
Third level education	13 (10.8)	Previously recorded extractions	115 (95.8)
Smoking	33 (27.5)	N° of teeth after procedures	16.8 ± 9.1
Diabetes	27 (22.5)	Teeth extracted per patient	4.6 ± 4.7
Kennedy’s class (superior arch)		Kennedy’s class (inferior arch)	
0	8 (6.7)	0	9 (7.5)
I	24 (20.0)	I	24 (20.0)
II	29 (24.2)	II	26 (21.7)
III	43 (35.8)	III	47 (39.2)
IV	1 (0.8)	IV	1 (0.8)
V	15 (12.5)	V	13 (10.8)

Data expressed as “number (%)” for categorical variables and “mean ± standard deviation” for continuous variables.

**Table 2:** Clinical variables stratified by smoking habits, gender and diabetes.

	All Patients n = 120	Non-Diabetic Patients n = 93	Diabetic Patients n = 27	*p* Value	Non Smoker n = 87	Smoker n = 33	*p* Value	Female n = 53	Male n = 67	*p* Value
Age	63.3 ± 15.8	61.0 ± 16.7	71.2 ± 9.0	*0.003*	66.9 ± 14.1	53.7 ± 16.3	*<0.001*	-	-	*-*
Sex (male)	67 (55.8)	51 (54.8)	16 (59.3)	*ns*	44 (50.5)	23 (69.7)	*ns*	-	-	*-*
Education level	-	-	-	*0.003*	-	-	*ns*	-	-	*ns*
Elementary school	31 (25.8)	19 (20.4)	12 (44.4)	*-*	27 (31.0)	4 (12.1)	*-*	16 (30.2)	15 (22.4)	*-*
Middle school	35 (29.2)	24 (25.8)	11 (40.7)	*-*	24 (27.6)	11 (33.3)	*-*	11 (20.7)	24 (35.8)	*-*
High school	41 (34.2)	38 (40.9)	3 (11.1)	*-*	28 (32.2)	13 (39.4)	*-*	19 (35.8)	22 (32.8)	*-*
Third level education	13 (10.8)	12 (12.9)	1 (3.7)	*-*	8 (9.2)	5 (15.1)	*-*	7 (13.2)	6 (8.9)	*-*
Previously recorded extractions	115 (95.8)	88 (94.6)	27 (100)	*ns*	83 (95.4)	32 (97.0)	*ns*	49 (92.4)	66 (98.5)	*ns*
N° of teeth before procedures	21.5 ± 7.5	22.5 ± 7.4	18.0 ± 6.8	*0.005*	21.2 ± 7.4	22.2 ± 7.6	*ns*	22.0 ± 7.2	21.0 ± 7.7	*ns*
N° of teeth after procedures	16.8 ± 9.1	17.8 ± 9.4	13.3 ± 7.15	*0.02*	14.6 ± 8.6	16.5 ± 10.3	*ns*	19.0 ± 8.4	15.1 ± 9.3	*0.02*
Teeth extracted per patient	4.6 ± 4.7	4.6 ± 5.1	4.7 ± 3.2	*ns*	3.8 ± 4.1	6.7 ± 5.6	*0.002*	3.0 ± 2.5	5.9 ± 5.6	*<0.001*

Data expressed as “number (%)” for categorical variables and “mean ± standard deviation” for continuous variables.

**Table 3:** Risk factors for tooth extraction.

**Risk Factors for Tooth Extraction**		
Risk factor	Univariate linear regression models coefficients and 95% CI (beta coefficient, 95% Confidence Interval)	*p* value
Age	0.02 (−0.03–0.07)	*p = 0.4*
Male Sex	2.89 (1.26–4.53)	*p = 0.001*
Smoke	2.95 (1.12–4.79)	*p = 0.002*
Diabetes	0.11 (−1.93–2.16)	*p = 0.9*
**Risk Factors for a Lower Number of Teeth before Extraction**		
Risk factor	Univariate linear regression models coefficients and 95% CI (beta coefficient, 95% Confidence Interval)	*p* value
Age	−0.24 (−0.31–−0.16)	*p < 0.001*
Male Sex	−0.95 (−3.68–1.77)	*p = 0.5*
Smoke	1.07 (−1.96–4.09)	*p = 0.5*
Diabetes	−4.47 (−7.61–−1.33)	*p = 0.006*

**Table 4:** Causes for extraction stratified for risk factors.

Causes for Extraction	All Extracted Teeth n = 554	Non-Diabetic Patients n = 427	Diabetic Patients n = 127	*p* Value	Non Smoker n = 331	Smoker n = 223	*p* Value	Non Smoker and/or Non Diabetic n = 510	Smoker and Diabetic n = 44	*p* Value
Caries	289 (52.2)	222 (52.0)	67 (52.8)	*0.04*	172 (52.0)	117 (52.5)	*ns*	268 (52.5)	21 (47.7)	*ns*
Periodontitis	198 (35.7)	146 (34.2)	52 (40.9)		120 (36.2)	78 (35.0)		176 (34.6)	22 (50.0)	
Prosthetic indication	16 (2.9)	15 (3.5)	1 (0.8)		7 (2.1)	9 (4.0)		16 (3.2)	0 (0)	
Endodontic lesion	38 (6.9)	35 (8.2)	3 (2.4)		21 (6.4)	17 (7.6)		37 (7.2)	1 (2.3)	
Failure of previous treatment	13 (2.3)	9 (2.1)	4 (3.1)		11 (3.3)	2 (0.9)		13 (2.5)	0 (0)	

Data expressed as “number (%)” for categorical variables; ns: non-significant.
